# Enzyme-assisted extraction of anti-inflammatory compounds from habanero chili pepper (*Capsicum chinense*) seeds

**DOI:** 10.3389/fnut.2022.942805

**Published:** 2022-09-08

**Authors:** Hector Emmanuel Cortes-Ferre, Marilena Antunes-Ricardo, Janet Alejandra Gutiérrez-Uribe

**Affiliations:** ^1^Tecnologico de Monterrey, Centro de Biotecnología-FEMSA, Monterrey, Mexico; ^2^Tecnologico de Monterrey, The Institute for Obesity Research, Monterrey, Mexico; ^3^Departamento de Ciencias, Tecnologico de Monterrey, Puebla, Mexico

**Keywords:** enzymatic treatment, chili pepper seeds, anti-inflammatory, phenolics, extraction kinetics, capsaicinoids, capsaicin

## Abstract

Capsaicinoids are the main bioactive compounds extracted from chili pepper seeds (CPSs) but other bioactive compounds such as phenolic compounds may be found. Enzyme-assisted extraction (EAE) improves the extraction of bioactive compounds from fruits and seeds. The aim of this study was to establish the cellulase-assisted extraction conditions of capsaicinoids and phenolic compounds from Habanero CPSs (*Capsicum chinense*) and to evaluate the anti-inflammatory activity of the obtained extracts on murine macrophages. EAE was performed using different temperatures (T1 = 30°C, T2 = 45°C and T3 = 60°C), enzyme concentrations (E1 = 2,500 UI/L and E2 = 250 UI/L), and extraction time periods (0-150 min). Total phenolic compounds were quantified using the Folin-Ciocalteu assay, capsaicin (CAP) and dihydrocapsaicin (DHC) contents were evaluated by HPLC, and anti-inflammatory activity was performed with Griess assay on murine macrophage RAW 264.7 cell culture. The highest phenolic compound content (337.96 mg GAE/L) was achieved at 30°C, 2,500 UI/L, and 150 min of extraction. The highest CAP content (310.23 μg/ml) was obtained at 45°C with 250 UI/L for 150 min, while for DHC (167.72 μg/ml), the conditions were 60°C, 2,500 UI/L, and 120 min. The highest anti-inflammatory response was obtained when 60°C, E2, and 150 min were used for the extraction, and nitric oxide (NO) production was reduced to 22.56%. Based on the results obtained in this research, EAE allowed the recovery of compounds with anti-inflammatory activity from CPS using water as a solvent. There was a correlation between the extraction of CAP and DHC. But although a moderate direct correlation between the concentration of capsaicinoids and total phenolic compounds (TPCs) and an inverse correlation of the presence of the bioactive compounds (TPC, CAP, and DHC) with the NO synthesis, these were not statistically significant. We demonstrated that Habanero seeds are an important raw material to recover anti-inflammatory compounds beyond capsaicinoids using water in EAE.

## Introduction

The chili pepper-related industry represents one of the main agricultural gross domestic products in those countries where this fruit is significant for their gastronomy and other cultural reasons. The exportation of this condiment follows strict commercial rules that unfortunately derivatives in an important amount of waste product that could be used as by-products due to their content of compounds with several bioactive properties (1). More than 20% of the pepper fruits for industrial purposes are discarded as by-products without any profitable worth (2). Most of the studies dedicated to analyze the red pepper bioactive components that have been focused on the pulp of the fruit, but its seeds may be used to obtain extracts that reduced pro-inflammatory cytokines and triglycerides in the blood (3). Jalapeño pepper seeds, considered a by-product of the scalding industry, are an important source of antioxidants (4) but greener methods of extraction are required to scale up the reuse of this food waste.

Capsaicin (CAP) is the main constituent of chili pepper and it causes the pungency of the fruit (5). This chemical compound has been described as an efficient anti-inflammatory compound in metabolic studies (6). CAP has the capability to link to transient receptor potential vanilloid (TRPV-1) that produces an intracellular signaling torrent when it is activated (7). CAP, *via* linking with TRPV-1, constrains the discharge of inflammatory mediators such as nitric oxide (NO) and pro-inflammatory cytokines, including TNF-α, IL-1β, and IL-6, in LPS-stimulated BV-2 microglia (8).

Capsaicinoids are the main bioactive compounds extracted from chili pepper seeds (CPSs) but other bioactive compounds such as flavonoids glycosides and other phenolic compounds may be found. Some authors such as Materska and Perucka (9) and Alam et al. (10) showed that flavonoids and other phenolics from CPSs could act on synergy to provide bioactive effects on human health such as antioxidant, antimutagenic, and anti-obesity. Cho et al. (11) described interesting health benefits such as antioxidant, antidiabetic, anti-obesity, anticarcinogenic, and anti-inflammatory properties from the chili pepper extracts from leaves and pulp; they found that in addition to CAP, luteolin glycosides and other phenolic compounds could be responsible for these bioactivities. In all these studies, bioactive compounds have been extracted using solvents different from water. Therefore, it is important to develop greener extraction methods.

**FIGURE 1:**
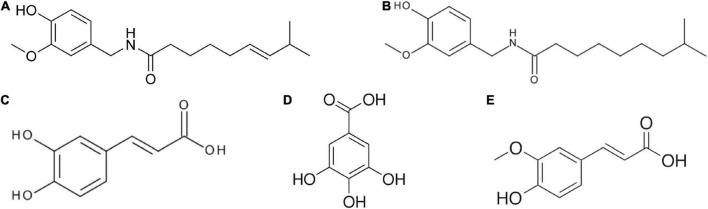
Chemical structure of bioactive compounds found in chili pepper extracts: **(A)** capsaicin (CAP), **(B)** dihydrocapsaicin (DHC), **(C)** caffeic acid, **(D)** gallic acid, and **(E)** ferulic acid.

The characteristic shape of CPSs is planar, like a disc with a specific vesicular depression in the center. The color of the seeds is dry yellow, and in most species, the seeds contain many essential fatty acids, complex carbohydrates such as cellulose and pectin, vitamins, nutrients, and bioactive phytochemicals such as phenolic and antioxidant compounds (12). Chouaibi et al. (13) analyzed the red pepper seed oil and found that the phenolic compound profile included gallic, caffeic, and ferulic acids (Figure 1).

Enzymatic-assisted extraction (EAE) has been used in many fruit and seed products (14). Chouaibi et al. (13) described the chemical composition of red pepper seeds and establish that more than 43% are cellulose complex that forms the cell wall. Additionally, Zhang et al. (15) explained that when raw seeds from chili pepper are solar or industrial dried, the ratio of cellulose and hemicellulose to xylan contents increases. These complex macromolecules in the cell wall of CPSs require the use of an enzymatic complex composed mainly of cellulase that could break the chemical composition and liberate the internal components. Capsaicinoids, flavonoids, tocopherols, and others are embedded into the cell wall (14, 16–18).

For this purpose, the aim of this study was to establish the extraction conditions of capsaicinoids and phenolic compounds from Habanero CPSs (*Capsicum chinense*) using cellulase-assisted extraction. The anti-inflammatory activity of the extracts was tested in a murine macrophage cell line (RAW 264.7) to confirm the bioactivity of the compounds recovered from CPS and their correlation with capsaicinoids or total phenolic compounds.

## Materials and methods

### Materials

Habanero CPSs (*Capsicum chinense*) were obtained from Italmesa Company, Puebla, Mexico. The enzymatic commercial complex Celuzyme (Cellulase, 121,000 UI/kg) was kindly donated by Enmex S.A. de C.V., Estado de Mexico, Mexico. Gallic acid and CAP (> 97%, M2028) used as standard in the HPLC quantification were purchased from Sigma (St. Louis, MO, United States). HPLC grade methanol and analytical grade formic acid were procured from Merck (Merck, India). For the *in vitro* experiments, Dulbecco’s Modified Eagle Medium (DMEM; Gibco, Gaithersburg, MD, United States), fetal bovine serum (GenDEPOT, Barker, TX, United States), and penicillin-streptomycin antibiotics were purchased. Finally, a murine macrophage cell line (RAW 264.7) was obtained from the American Type Cell Collection (ATCC, Manassas, VA, United States).

### Methods

#### Enzymatic pretreatment

Habanero seeds were mixed with the enzyme solution in a 1:15 (w:v) proportion and incubated at a controlled temperature water bath (Branson Bransonic^®^ CPXH Digital Bath 3800, Emerson, St. Louis, MO, United States). Enzymatic pretreatment extraction kinetics were performed using different temperatures (T1 = 30°C, T2 = 45°C, and T3 = 60°C) and enzyme concentrations (E1 = 2,500 UI/L and E2 = 250 UI/L) based on previous reports (16–18). Extraction was performed for 150 min, and every 30 min, a sample was taken. Once the processing time was over, the supernatant was separated from the extract, and the CPS extract was stored at -20°C until use.

#### Quantification of total phenolic compounds

The quantification of total phenolic compounds (TPCs) was performed by following the methodology proposed by Medina-Remón et al. (19). In brief, the sample was prepared by mixing 1 ml of CPS extract, 1 ml of Ultrapure water (Milli-Q), and 34 μl of HCl (35% v/v). For the 96-well plate preparation, each well contained 15 μl of the diluted sample, 170 μl of ultrapure water (Milli-Q), 12 μl of Folin-Ciocalteu reagent, and 30 μl of sodium carbonate. The mixtures were incubated for 1 h at room temperature in the dark. After the reaction period, 73 μl of Milli-Q water were added with the multichannel pipette. Absorbance was measured at 765 nm using a UV/VIS spectrophotometer (BioTek ELx800, BioTek Instruments, Winooski, VT, United States). The TPC content was expressed as mg gallic acid equivalent (GAE) per gram of extract using a calibration curve, and then, the increase was calculated based on the content at *t* = 0 min.

#### Capsaicin quantification

The identification and quantification of CAP were performed according to the methodology of Othman et al. (20). In brief, after extraction from CPSs, the supernatant was filtered through a 0.45 μm nylon filter into an HPLC sample vial using a disposable syringe. The chromatographic analysis of CAP and dihydrocapsaicin (DHC) was performed using high-performance liquid chromatography (HPLC) (Agilent 1100 Series Santa Clara, CA, United States) with a Zorbax Eclipse Plus C_18_ (particle size 4.6 × 100 mm 3.5 μm) column at 40°C, a flow of 0.8 ml/min, and an injection volume of 10 μl. The mobile phase consisted of (A) HPLC-grade water with 0.1% of formic acid and (B) HPLC-grade methanol. The gradient started with 50% of B for 2 min, increasing to 70% in 5 min, and in the next 5 min, the percentage of B changed to 80%. After 15 min, the percentage of B increased to 100% and remained in an isocratic mode for another 5 min. Chromatograms were obtained at 280 nm. The standard curve for CAP was used (*y* = 4.9188*x* + 5.7049; *R*^2^ = 0.9999). The CAP and DHC concentrations in samples were expressed as μg of CAP equivalents/ml of extract.

#### Cell culture

Murine macrophage cell line (RAW 264.7) was maintained in Dulbecco’s Modified Eagle’s Medium (DMEM) supplemented with 5% fetal bovine serum (FBS) with 1% penicillin/streptomycin at 37°C in a humidified atmosphere containing 5% CO_2_ until use.

#### Cytotoxicity and nitric oxide assays

The cytotoxicity assay was performed using the modification suggested by Bhatta et al. (21) using the CellTiter 96^®^ Aqueous One Solution Cell Proliferation Assay Kit (Promega, Madison, WI, United States), according to the manufacturer’s instructions. In brief, 100 μl were inoculated at a density of 1 × 10^4^ cells per well into 96-well plates and cultured at 37°C for 24 h. The cells were pretreated with 50 μl of CPS extracts at 0.01% (v/v) for 48 h before adding 50 μl of growth media (for control wells) or 50 μl LPS (10 μg/ml). After 48 h of incubation, 100 μl were removed for NO assay, and into the remaining volume, 10 μl of CellTiter 96 AQueous One Solution reagent was added to each well to perform the cytotoxicity assay. The cells were incubated for 1 h, and absorbance at 490 nm was read using a microplate reader (BioTek ELx800, BioTek Instruments, Winooski, VT, United States). The absorbance was directly proportional to the number of living cells, and viability was expressed as a percent relative to control (untreated cells).

Nitrite accumulated in the macrophage culture medium was measured as an estimation of NO, and the cell culture supernatants were obtained from the 96-well plates used for the cytotoxicity assay. NO in cell-free culture supernatants was measured using Griess reagent (22) according to Cione et al. (23). In brief, cell culture media (100 μl) reacted with Griess System (Promega, Madison, WI, United States) reagents according to the kit manufacturer (20 μl reagent A and 20 μl of reagent B) incubated at room temperature for 10 min for each reagent, and then, absorbance was measured at 540 nm using a microplate reader (BioTek ELx800, BioTek Instruments, Winooski, VT, United States).

#### Statistical analyses

Data were expressed as means ± standard deviation of at least three independent experiments unless otherwise indicated. All variables were analyzed using ANOVA followed by a *post hoc* Tukey multiple comparison test (*p* < 0.05). All response variables were summited to a multivariate correlation with factorial analysis of principal components (*p* < 0.05), and these analyses were processed by the statistical analysis software SAS JMP (SAS Institute Inc., Cary, NC, United States).

## Results and discussion

### Extraction yield

**FIGURE 2:**
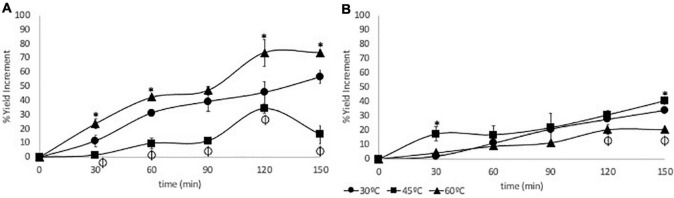
Extraction yield increase at different enzyme-assisted extraction (EAE) time periods using an enzyme concentration of 2,500 UI/L **(A)** or 250 UI/L **(B)**. (*) Highest increase at each sampling time after Tukey statistical test, and (ϕ) lowest increase at each sampling time after Tukey statistical test.

The extraction yield increased with the time of extraction (*p* < 0.0001), and it was positively affected by the process temperature (*p* < 0.0001), although the interaction of both factors was not statistically significant (*p* = 0.0679). The highest yield increment (73.5%) was achieved when the extraction was performed at 60°C during 150 min of the process with high enzyme concentration (E1). The lowest yield increment (1.42%) was obtained at 45°C with 30 min of extraction using high enzyme concentration (Figure 2A). Compared with conventional extraction techniques, EAE improved the extraction yield due to the direct activity through the seed cell wall, and it is potentially recognized as a green extraction technique for the use of water as a solvent (24). Magaña-Barajas et al. reported that for ethanolic conventional extraction of CAP from *Capsicum chinense*, the extraction yield reached 40%, our results show that using high enzyme concentration at 60°C with 150 min of the process shows an extraction yield of 73% demonstrating that the use of this novel technique could improve the recovery of bioactive compounds (25). Farias et al. used cellulase enzymatic complex to macerate Tabasco pepper in proportions 0:15, 1:15, and 5:15 and found that the extraction yield increased by 17.5% with respect to conventional methods showing the potential of this technique (26).

The percentage of yield increment was almost twice as higher when using high enzyme concentration than the obtained with the low concentration (Figure 2B). In contrast to the high enzyme concentration, the yield increment did not reach a maximum value. Surendran et al. demonstrated that the presence of phenolic compounds such as salicylic acid, ferulic acid, gallic acid, and vanillin shut down the cellulose activity and therefore affected the extraction yield (27).

### Total phenolic compound content in capsaicin extracts

The extraction of phenolic compounds changed depending on the temperature of extraction (*p* < 0.0001), the enzyme concentration (*p* = 0.0009), and increased with the extraction time (*p* < 0.0001) (Table 1). The highest TPC increase (79-87%) was obtained at 30°C with 120 or 150 min of extraction using high enzyme concentration. At the low enzyme concentration (E2), an increase of 58% was obtained after 120 min of hydrolysis at 30°C, reaching 14.87 mg GAE/g.

**TABLE 1:** Total phenolic concentration in chili pepper seed (CPS) extracts obtained at different time periods, temperatures, and cellulase concentrations.

Enzyme concentration [UI/L]	T (°C)	Total phenolic concentration [mg GAE/g] at different EAE time (min)				
		30	60	90	120	150
2500	30	12.04 (11%) ^k,l^	11.65 (27%) ^g,h,i^	12.63 (46%) ^c,d,e^	14.87 (79%) ^a^	14.38 (87%) ^a^
	45	11.93 (6%) ^k,l^	10.04 (6%) ^k,l^	11.70 (13%) ^j,k,l^	11.33 (17%) ^i,j,k,l^	12.41 (18%) ^i,j,k^
	60	11.94 (15%) ^j,k,l^	11.23 (24%) ^h,i,j^	12.26 (40%) ^e,f^	12.87 (62%) ^b^	11.68 (58%) ^b,c^
250	30	12.77 (9%) ^k,l^	12.78 (12%) ^j,k,l^	13.12 (30%) ^f,g,h^	14.87 (58%) ^b,c^	13.88 (59%) ^b^
	45	11.77 (5%) ^l^	12.38 (6%) ^k,l^	12.63 (17%) ^i,j,k,l^	11.25 (22%) ^h,i,j^	12.37 (33%) ^f,g,h^
	60	11.54 (27%) ^g,h,i^	12.10 (39%) ^e,f,g^	11.79 (45%) ^d,e^	11.47 (48%) ^b,c,d,e^	11.41 (55%) ^b,c,d^

() Values in parenthesis indicate the increase as a percentage of total phenolic concentration of CPS extracts compared with the initial time. ^a^Different letters indicate significant differences in each panel after Tukey statistical test (p < 0.05).

In this study, TPCs in CPS extracts were higher than those reported by Gurnani et al. (28) for n-hexane and chloroform extracts from Indian red CPSs with TPC contents of approximately 7.5-26.15 mg GAE/g. Liu et al. (29) reported high total phenolic content in CPSs (3.93-6.21 mg GAE/g) using methanol, ethanol, or acetone but no information about the yield was described. Grimaldi et al. characterized extracts from *Capsicum annum* fruits dried at different temperatures (45-65°C) and showed that the TPC decreased meanwhile the temperature increased from 6.9 mg GAE/g (45°C) to 6 mg GAE/g (65°C), in concordance with our results that the TPC equally decreased with the increasing of the temperature of extraction due the oxidation of phenolic compounds (30). Regarding seeds, Leng et al. showed the total phenolic content of Australian growth bell pepper seeds and demonstrated that the variety of bell pepper did not affect the phenolic contents of extracts from the seeds (0.43-0.53 mg GAE/g), in contrast with our results in this research demonstrated that the use of EAE improved significantly the TPC of CPS extracts (31). These results demonstrate that EAE is a green alternative to recover phenolic compounds from CPS.

### Capsaicinoid quantification

**FIGURE 3:**
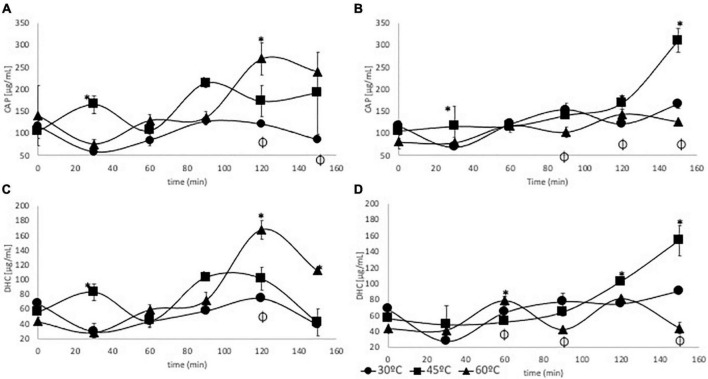
Extraction kinetics of **(A)** CAP with an enzyme concentration of 2,500 UI/L, **(B)** CAP with an enzyme concentration of 250 UI/L, **(C)** DHC with an enzyme concentration of 2,500 UI/L, and **(D)** DHC with an enzyme concentration of 250 UI/L. (*) Highest increase at each sampling time after Tukey statistical test, and (ϕ) lowest increase at each sampling time after Tukey statistical test.

Enzyme concentration did not have a significant effect on the extraction yield of CAP or DHC, contrary to temperature or extraction time (*p* < 0.05). The highest concentration of CAP (268.60 μg/ml) was reached after 120 min of hydrolysis at 60°C and high enzyme concentration (Figure 3A). No significant change in CAP concentration was observed during the 150 min of EAE at 30°C, and less than 121.01 μg/ml was reached. When EAE was performed at 60°C, there was a significant reduction in the CAP and DHC contents at 150 min compared with 120 min. Preliminary reports showed that at 60°C, CAP retention in Habanero seeds was reduced by 66% due to the disruption of a “protective layer” that causes high preservation of this compound (32).

For the low enzyme concentration (Figure 3B), the best results were achieved at 45°C and 150 min of EAE, reaching a CAP concentration of 310.23 μg/ml that was 47 and 60% higher than the obtained at 30 and 60°C, respectively. Santamaría et al. (17) found that, at 50°C, the CAP yield was better than that obtained at 80°C using commercial enzymatic complexes such as Celluclast, Viscozyme L, and Olivex for EAE from Guajillo chili flour. According to the author, after the enzymatic action over the cellulosic cell wall, the remaining cutin hinders the deeper action of the enzymatic treatment and reduces the release of bioactive compounds.

For the DHC extraction with high enzyme concentration (Figure 3C), the highest concentration levels were achieved at 120 min, and at 60°C, the concentration (167.72 μg/ml) was 66 and 40% higher than the ones achieved at 30 and 45°C, respectively. Salgado-Roman et al. (18) found that at extraction time between 70 to 90 min, the concentrations of capsaicinoids, carotenoids, and soluble sugars were higher than those obtained at 100 min using enzymatic pretreatment with chili pepper at 33°C. At low enzyme concentration (Figure 3D), DHC concentration (153.57 μg/ml) obtained at 45°C and 150 min was significantly higher than the concentrations achieved at 30 and 60°C.

### Effect of the CPS extracts on cell cytotoxicity and NO production in LPS-stimulated macrophages

**FIGURE 4:**
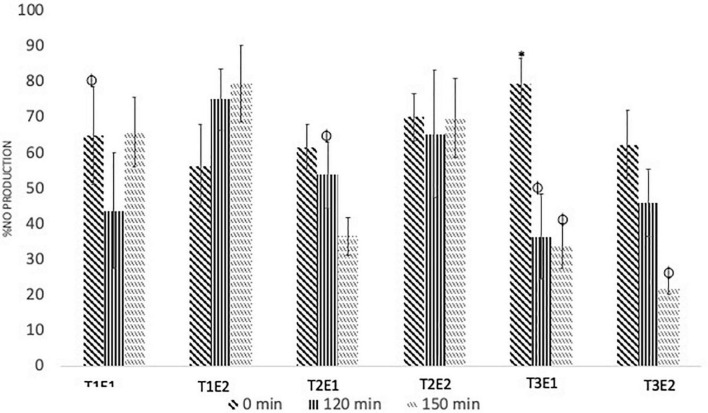
Relative nitric oxide (NO) production (%) observed for CPS extracts obtained after enzymatic treatment at different combinations of temperature (T1 = 30°C, T2 = 45°C, and T3 = 60°C) enzymes (E1 = 2,500 UI/L, E2 = 250 UI/L) and time periods (0, 120 and 150 min) (*) Highest relative production for the same combination of temperature and enzyme after Tukey statistical test, and (ϕ) lowest relative production for the same combination of temperature and enzyme after Tukey statistical test.

**FIGURE 5:**
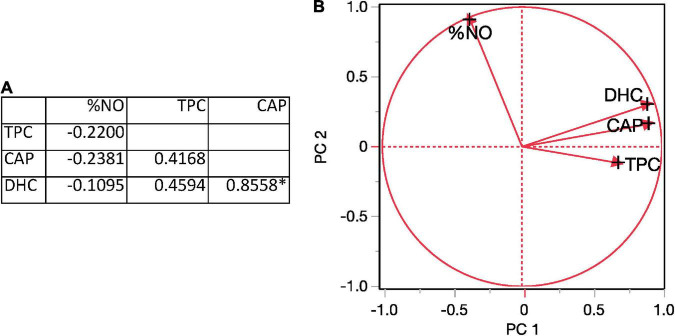
Correlation analysis of the extraction variables: **(A)** correlation matrix for the total phenolic compound (TPC), CAP, DHC, and %NO production, and **(B)** factor loading plot of principal components of the extraction variables.

Chili pepper seed extracts did not affect the growth of the macrophage cells when tested at 0.01% v/v (data not shown). As was observed with capsaicinoids, the enzyme concentration used to obtain the CPS extracts did not have a significant effect on the reduction of LPS-induced NO production in macrophages. At 30°C, only the CPS extract obtained at 120 min with the high enzyme concentration significantly reduced the NO production compared with that obtained at the initial time. When EAE was performed at 45°C, the only CPS extract that reduced the NO production was obtained at 150 min with a high enzyme concentration. Also, at the high enzyme concentration but at 60°C, the CPS extract obtained at 120 and 150 min showed lower NO production than that obtained in the beginning of the hydrolysis. At 60°C, even with a low enzyme concentration, the CPS extract obtained after 150 min of hydrolysis showed the best inhibitory effects on NO production (Figure 4). The reduction in NO production of CPS extracts was not correlated with the TPC or capsaicinoid content (Figure 5A). Principal component 1 from PCA analysis was mainly related to the bioactive compositions of the extracts, and principal component 2 was associated with the bioactivity of the CPS extracts. PCA showed that if the bioactive compound content rises, then the anti-inflammatory activity grows as well. Qiao et al. showed that there was no positive correlation between anti-inflammatory activity and the concentration of bioactive compounds in chili pepper extracts (33). CAP concentration showed a significant correlation (*p* < 0.05) with DHC concentration and a moderate but not statistically significant correlation with phenolic compound content (Figure 5B).

## Conclusion

We demonstrated that EAE is a green method to recover bioactive compounds from CPS. The highest total phenolic content was achieved at 30°C after 150 min of extraction using the high enzyme concentration while the highest concentration of CAP was obtained at 45°C using a low concentration of enzyme (250 UI/L) at 150 min of extraction. Regarding the evaluation of the anti-inflammatory activity, the best effect was observed with the extract obtained at 60°C using low enzyme concentration and 150 min. No correlation of TPC or CPS was found with the anti-inflammatory activity, and further experiments are required to identify phenolic compounds and their interactions with capsaicinoids.

## Data availability statement

The raw data supporting the conclusions of this article will be made available by the authors, without undue reservation.

## Author contributions

HC-F, MA-R, and JG-U designed the research and wrote the manuscript. All authors contributed to the article and approved the submitted version.

## References

[1] Cortés-Ferré HE Guajardo D Gutiérrez-Uribe JA. Recovery of capsaicinoids and other phytochemicals involved with TRPV-1 receptor to re-valorize chili pepper waste and produce nutraceuticals. *Front Sustain Food Syst.* (2021) 4:588534. 10.3389/fsufs.2020.588534

[2] Fornereto Soldan AC Arvelos S Watanabe ÉO Hori CE. Supercritical fluid extraction of oleoresin from *Capsicum annuum* industrial waste. *J Clean Prod.* (2021) 297:126593. 10.1016/J.JCLEPRO.2021.126593

[3] Kim JH Jin CH. Inhibitory activity of flavonoids, chrysoeriol and luteolin-7-o-glucopyranoside, on soluble epoxide hydrolase from *Capsicum* Chinense. *Biomolecules.* (2020) 10:180. 10.3390/biom10020180 31991570 PMC7072517

[4] Sandoval-Castro CJ Valdez-Morales M Oomah BD Gutiérrez-Dorado R Medina-Godoy S Espinosa-Alonso LG. Bioactive compounds and antioxidant activity in scalded Jalapeño pepper industrial byproduct (*Capsicum annuum*). *J Food Sci Technol.* (2017) 54:1999–2010. 10.1007/s13197-017-2636-2 28720957 PMC5495727

[5] Tobolka A Škorpilová T Dvoøáková Z Cusimamani EF Rajchl A. Determination of capsaicin in hot peppers (*Capsicum* spp.) by direct analysis in real time (DART) method. *J Food Compos Anal.* (2021) 103:104074. 10.1016/J.JFCA.2021.104074

[6] Panchal SK Bliss E Brown L. Capsaicin in metabolic syndrome. *Nutrients.* (2018) 10:14–8. 10.3390/nu10050630 29772784 PMC5986509

[7] Ilie M Caruntu C Tampa M Georgescu S-R Matei C Negrei C Capsaicin: physicochemical properties, cutaneous reactions and potential applications in painful and inflammatory conditions (review). *Exp Ther Med.* (2019) 18:916–25. 10.3892/etm.2019.7513 31384324 PMC6639979

[8] Bassi MS Gentile A Iezzi E Zagaglia S Musella A Simonelli I Transient receptor potential vanilloid 1 modulates central inflammation in multiple sclerosis. *Front Neurol.* (2019) 10:30. 10.3389/fneur.2019.00030 30761069 PMC6361812

[9] Materska M Perucka I. Antioxidant activity of the main phenolic compounds isolated from hot pepper fruit (*Capsicum annuum* L.). *J Agric Food Chem.* (2005) 53:1750–6. 10.1021/jf035331k 15740069

[10] Alam MA Saleh M Mohsin GM Nadirah TA Aslani F Rahman MM Evaluation of phenolics, capsaicinoids, antioxidant properties, and major macro-micro minerals of some hot and sweet peppers and ginger land-races of Malaysia. *J Food Process Preserv.* (2020) 44:e14483. 10.1111/jfpp.14483

[11] Cho SY Kim HW Lee MK Kim HJ Kim JB Choe JS Antioxidant and anti-inflammatory activities in relation to the flavonoids composition of pepper (*Capsicum annuum* L.). *Antioxidants.* (2020) 9:1–11. 10.3390/antiox9100986 33066301 PMC7602036

[12] Jahan N Rahman K. Chapter 39: Cold pressed capia pepper (*Capsicum annuum* L.) seed oil. In: Ramadan M. F. editor. *Cold Pressed Oils*. Cambridge, MA: Academic Press (2020). p. 439–47.

[13] Chouaibi M Rezig L Hamdi S Ferrari G. Chemical characteristics and compositions of red pepper seed oils extracted by different methods. *Ind Crops Prod.* (2019) 128:363–70. 10.1016/j.indcrop.2018.11.030

[14] Batiha GES Alqahtani A Ojo OA Shaheen HM Wasef L Elzeiny M Biological properties, bioactive constituents, and pharmacokinetics of some *Capsicum* spp. And capsaicinoids. *Int J Mol Sci.* (2020) 21:1–35. 10.3390/ijms21155179 32707790 PMC7432674

[15] Zhang R-Y Liu H-M Ma Y-X Wang X-D. Effects of roasting on composition of chili seed and storage stability of chili seed oil. *Food Sci Biotechnol.* (2019) 28:1475–86. 10.1007/s10068-019-00578-9 31695946 PMC6811469

[16] Gamarra Mendoza N Velasquez Rodriguez S Roque Lima B. Improvement of the extraction of carotenoids and capsaicinoids of chili pepper native (*Capsicum baccatum*), assisted with cellulolytic enzymes. *Rev Peru Biol.* (2020) 27:55–060. 10.15381/rpb.v27i1.17588

[17] Santamaría RI Reyes-Duarte MD Bárzana E Fernando D Gama FM Mota M Selective enzyme-mediated extraction of capsaicinoids and carotenoids from chili guajillo puya (*Capsicum annuum* L.) using ethanol as solvent. *J Agric Food Chem.* (2000) 48:3063–7. 10.1021/jf991242p 11032487

[18] Salgado-Roman M Botello-Álvarez E Rico-Martínez R Jiménez-Islas H Cárdenas-Manríquez M Navarrete-Bolaños JL. Enzymatic treatment to improve extraction of capsaicinoids and carotenoids from chili (*Capsicum annuum*) fruits. *J Agric Food Chem.* (2008) 56:10012–8. 10.1021/jf801823m 18847207

[19] Medina-Remón A Barrionuevo-González A Zamora-Ros R Andres-Lacueva C Estruch R Martínez-González MÁ Rapid Folin-Ciocalteu method using microtiter 96-well plate cartridges for solid phase extraction to assess urinary total phenolic compounds, as a biomarker of total polyphenols intake. *Anal Chim Acta.* (2009) 634:54–60. 10.1016/j.aca.2008.12.012 19154810

[20] Al Othman ZA Ahmed YBH Habila MA Ghafar AA. Determination of capsaicin and dihydrocapsaicin in *Capsicum* fruit samples using high performance liquid chromatography. *Molecules.* (2011) 16:8919–29. 10.3390/molecules16108919 22024959 PMC6264681

[21] Bhatta P Dhukhwa A Sheehan K Al Aameri RFH Borse V Ghosh S Capsaicin protects against cisplatin ototoxicity by changing the STAT3/STAT1 ratio and activating Cannabinoid (CB2) receptors in the cochlea. *Sci Rep.* (2019) 9:1–16. 10.1038/s41598-019-40425-9 30858408 PMC6411993

[22] Granger DL Taintor RR Boockvar KS Hibbs JB. Measurement of nitrate and nitrite in biological samples using nitrate reductase and Griess reaction. *Methods Enzymol.* (1996) 268:142–51. 10.1016/s0076-6879(96)68016-1 8782580

[23] Cione E Plastina P Pingitore A Perri M Caroleo MC Fazio A Capsaicin analogues derived from n-3 polyunsaturated fatty acids (PUFAs) reduce inflammatory activity of macrophages and stimulate insulin secretion by β-cells in vitro. *Nutrients.* (2019) 11:915. 10.3390/nu11040915 31022842 PMC6520993

[24] Castro-Muñoz R Gontarek-Castro E Jafari SM. Up-to-date strategies and future trends towards the extraction and purification of Capsaicin: a comprehensive review. *Trends Food Sci Technol.* (2022) 123:161–71. 10.1016/J.TIFS.2022.03.014

[25] Magaña-Barajas E Buitimea-Cantúa GV Hernández-Morales A del Torres-Pelayo VR Vázquez-Martínez J Buitimea-Cantúa NE. In vitro α-amylase and α-glucosidase enzyme inhibition and antioxidant activity by capsaicin and piperine from *Capsicum* chinense and Piper nigrum fruits. *J Environ Sci Health B.* (2021) 56:282–91. 10.1080/03601234.2020.1869477 33397190

[26] de Farias VL da Silva Araújo ÍM da Rocha RFJ dos Garruti DS Pinto GAS. Enzymatic maceration of Tabasco pepper: effect on the yield, chemical and sensory aspects of the sauce. *LWT.* (2020) 127:109311. 10.1016/j.lwt.2020.109311

[27] Surendran A Siddiqui Y Ali NS Manickam S. Inhibition and kinetic studies of cellulose- and hemicellulose-degrading enzymes of Ganoderma boninense by naturally occurring phenolic compounds. *J Appl Microbiol.* (2018) 124:1544–55. 10.1111/JAM.13717 29405525

[28] Gurnani N Gupta M Mehta D Mehta BK. Chemical composition, total phenolic and flavonoid contents, and in vitro antimicrobial and antioxidant activities of crude extracts from red chilli seeds (Ca*psicum frutescens* L.). *J Taibah Univ Sci.* (2016) 10:462–70. 10.1016/J.JTUSCI.2015.06.011

[29] Liu Y Chen Y Wang Y Chen J Huang Y Yan Y Total phenolics, capsaicinoids, antioxidant activity, and α-glucosidase inhibitory activity of three varieties of pepper seeds. *Int J Food Prop.* (2020) 23:1016–35.

[30] Grimaldi M Cavazza A Pitirollo O Zoccali M Mondello L Giuffrida D Analytical evaluation of carotenoids, apocarotenoids, capsaicinoids, and phenolics to assess the effect of a protective treatment on chili peppers dried at different temperatures. *Eur Food Res Technol.* (2022) 248:2339–49. 10.1007/s00217-022-04049-0

[31] Leng Z Zhong B Wu H Liu Z Rauf A Bawazeer S Identification of phenolic compounds in australian-grown bell peppers by liquid chromatography coupled with electrospray ionization-quadrupole-time-of-flight-mass spectrometry and estimation of their antioxidant potential. *ACS Omega.* (2022) 7:4563–76. 10.1021/acsomega.1c06532 35155947 PMC8829910

[32] Palma-Orozco G Orozco-Álvarez C Chávez-Villeda AA Mixtega-Martínez A Castro-Muñoz R. Capsaicin content in red habanero chilli (*Capsicum chinense* Jacq.) and its preservation after drying process. *Future Foods.* (2021) 4:100070. 10.1016/J.FUFO.2021.100070

[33] Qiao GH Wexxin D Zhigang X Sami R Khojah E Amanullah S. Antioxidant and anti-inflammatory capacities of pepper tissues. *Ital J Food Sci.* (2020) 32:265–74. 10.14674/IJFS-1700

